# Design and validation of magnetic particle spectrometer for characterization of magnetic nanoparticle relaxation dynamics

**DOI:** 10.1063/1.4978003

**Published:** 2017-03-02

**Authors:** Nicolas Garraud, Rohan Dhavalikar, Lorena Maldonado-Camargo, David P. Arnold, Carlos Rinaldi

**Affiliations:** 1 Department of Electrical and Computer Engineering, University of Florida, Gainesville, Florida 32608, USA; 2 Department of Chemical Engineering, University of Florida, Gainesville, Florida 32608, USA; 3 J. Crayton Pruitt Family Department of Biomedical Engineering, University of Florida, Gainesville, Florida 32608, USA

## Abstract

The design and validation of a magnetic particle spectrometer (MPS) system used to study the linear and nonlinear behavior of magnetic nanoparticle suspensions is presented. The MPS characterizes the suspension dynamic response, both due to relaxation and saturation effects, which depends on the magnetic particles and their environment. The system applies sinusoidal excitation magnetic fields varying in amplitude and frequency and can be configured for linear measurements (1 mT at up to 120 kHz) and nonlinear measurements (50 mT at up to 24 kHz). Time-resolved data acquisition at up to 4 MS/s combined with hardware and software-based signal processing allows for wide-band measurements up to 50 harmonics in nonlinear mode. By cross-calibrating the instrument with a known sample, the instantaneous sample magnetization can be quantitatively reconstructed. Validation of the two MPS modes are performed for iron oxide and cobalt ferrite suspensions, exhibiting Néel and Brownian relaxation, respectively.

## I. INTRODUCTION

Magnetic particle spectrometer (MPS) development is motivated to assess magnetic suspension suitability for magnetic particle imaging (MPI).^1,2^ MPI is an emerging biomedical imaging technique addressing drawbacks found in nuclear imaging by using non-radioactive tracers, i.e. the magnetic nanoparticles, with theoretically higher resolution in a short process time. MPI detects nanoparticle density spatially by probing locally their dynamic magnetization in a spatial selection gradient field and finds application in real-time cardiovascular imaging,^3^ stem cell tracking^4,5^ and hyperthermia.^6^ The MPS described herein has no spatial scanning capability, but can assess both particle suspension relaxation and saturation, related to their performance for MPI.^7^

The designed system features the measurement of full time-series data,^8^ as opposed to discrete FFT components using a lock-in amplifier;^9^ multi-mode attenuation/cancellation^9^ of the primary excitation signal (99.7 % of the feed-through, i.e. -50 dB); and an estimation of the instantaneous magnetization of the suspension, instead of just the induced voltage.

The dynamic response of the magnetic suspension depends on the strength and frequency of the applied magnetic field. Nonlinearity in response increases with magnetic field amplitude because of particle magnetic saturation, while relaxation effects become more evident with increasing frequency. Observing and quantifying these phenomena is relevant to study the dynamic response of magnetic suspensions, to improve their synthesis, or to infer on their suitability for diverse applications, such as MPI.

The designed MPS can operate in two modes, herein referred to as “linear DMS” (dynamic magnetic susceptibility) and “nonlinear MPS”. At low applied field amplitudes, linear DMS probes only the linear magnetization regime of the nanoparticles, similarly to AC susceptometry. The response to a sinusoidal time-varying magnetic field is a sinusoidal magnetic moment change, from which the complex magnetic susceptibility, characteristic of the suspension rotational dynamics, can be determined. At higher applied field amplitudes, nonlinearity appears in the sample response due to magnetic saturation of the suspension. The magnetization saturates, yielding sharper, non-sinusoidal voltage changes when the magnetization flips. The measured spectrum presents odd harmonics of the fundamental frequency, characteristic of the suspension magnetic nonlinearity. This nonlinear MPS mode characterizes the nanoparticle suspension rotational dynamics, both in amplitude and in frequency, assessing both saturation and relaxation effects.

## II. MPS SETUP DESIGN

**FIG. 1.**
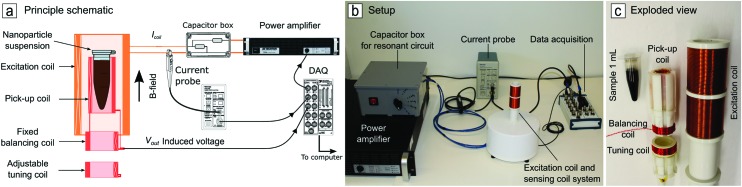
The nanoparticle suspension is driven by a time-varying magnetic field provided by the excitation field. The sample magnetization change induces a voltage in a pick-up coil system which is recorded by a DAQ.

The MPS provides a spatially uniform, sinusoidally time-varying magnetic field to a nanoparticle suspension in a 1 mL Eppendorf vial. The field is generated by a gapped solenoid excitation coil, with sinusoidal current supplied via a power amplifier fed by a computer-controlled data acquisition (DAQ) system (National Instruments PCI-6115, 12-Bit, 4 MS/s for multi-channel I/O) (Figure 1). The nanoparticles rotate in response to the applied magnetic field, inducing a change in magnetic flux detected by a pick-up coil system. The induced voltage and driving current are recorded simultaneously by the DAQ.

### A. Excitation field

The excitation coil dimensions (AWG 19, 206 turns, ∅ 30 mm, 73 mm long) are optimized to provide a large, homogeneous magnetic field (3 mT/A, 5 % inhomogeneity over the sample volume) while keeping its resistance and inductance low (0.58 Ω, 400 μH respectively), which allows more current to be provided by the power amplifier (AE Techron 7224, voltage gain of 20) at high frequencies. The DAQ outputs the sinusoidal excitation waveform to the power amplifier, and the excitation coil is either directly connected to the power amplifier terminals or connected via a resonant matching circuit in order to achieve higher field amplitudes. The direct connection mode provides wide-band measurements in the linear DMS mode, with maximum field amplitudes of 50 mT for frequencies up to 5 kHz, 10 mT up to 30 kHz, and 1 mT up to 120 kHz. The resonant matching circuit is implemented using pairs of high-voltage capacitors (Cornell-Dubilier) designed for a current gain of 3 at discrete frequencies in the nonlinear MPS mode. In this way, the system can reach 50 mT at 3, 10.8, 16, 19.6 and 24 kHz. Only measurements at 3 kHz and 24 kHz are presented in this paper using resonant matching circuits. The input excitation current shows -50 dBc/Hz phase noise at 1 Hz offset from the carrier (-70 dBc/Hz at 10 Hz offset) and total harmonic distortion of better than -63 dB across all cases, confirming high spectral purity of the excitation magnetic field.

### B. Signal measurement and feed-through cancellation strategy

The DAQ simultaneously measures the current delivered to the excitation coil and the nanoparticle response as measured by a pick-up coil system. The excitation coil current is measured by a current probe (Tektronix, TCP305A probe with TCPA300 amplifier) to assess the reference phase of the excitation magnetic field. A primary design challenge for the sensing coil system is to negate the direct induction from the excitation coil (called “feed-through”), and ideally only measure the magnetic moment of the nanoparticle suspension. To mitigate feed-through, the sensing coil system consists of three coils: the pick-up coil sensing the sample magnetization change, a fixed balancing coil, and an adjustable fine-tuning coil as illustrated on Figure 1. The sensing coil system has a rms noise of 0.32 mV, and a self-resonance frequency of 1 MHz, which sets the upper frequency bound for measurements.

The pick-up coil is internally molded in epoxy resin (AWG 28, 40 turns, ∅10.5 mm) to minimize the distance between the coil and the sample, thereby maximizing the sensitivity to the nanoparticle sample while minimizing feed-through. Next, a balancing coil is mounted in series with the pick-up coil and wound in opposite direction. The excitation field induces a voltage with opposite phase that counteracts the main induction from the pick-up coil. The balancing coil, which is very sensitive to any displacement, is fixed at 25 mm from the pick-up coil, to avoid interaction with the sample. Furthermore, to allow fine cancellation adjustments, a movable short-circuited fine-tuning coil is placed close to the fixed balancing coil. The fine-tuning coil, being inductively coupled to both the excitation and balancing coils, modifies the balancing and further reduces the feed-through.

### C. Signal processing for determination of instantaneous magnetization

For the data reported here, the first 0.25 s of all measurements are discarded to eliminate any transients from the electronics. For the linear mode, the sampling rate is set at approximately 30 times the excitation frequency (the DAQ imposes discrete sampling frequencies) and up to 5 s of data is used (minimum of 2500 cycles). For the nonlinear mode, 1 s of data is used (minimum of 3000 cycles) with a sampling rate ≥100 times the excitation frequency so that at least 50 harmonics can be extracted.

In addition to the sensing coil system, which minimizes but does not fully eliminate feed-through at the hardware level, additional feed-through is cancelled numerically during the post-processing. Two measurements are performed sequentially without ($v_{blank}\left(t\right)$) and with ($v_{sample}\left(t\right)$) the sample present. The fast Fourier transform (FFT) is applied to both signals (bin width in the FFT spectrum is approximately 1 Hz in all cases) generating frequency-domain spectra ${\tilde{V}}_{blank}\left(f\right)$ and ${\tilde{V}}_{sample}\left(f\right)$, each represented by phasor amplitudes *A*_*j*_ and phases $\varphi_{j}$. From here on, only the FFT coefficients at the excitation frequency *f*_0_ and subsequent even and odd harmonics are considered; all other FFT coefficients are discarded, which acts to filter the relevant signal information. Next, ${\tilde{V}}_{suspension}$ is obtained by subtracting ${\tilde{V}}_{blank}$ from ${\tilde{V}}_{sample}$.^10^ The time-domain voltage induced by the suspension is then reconstructed, which is also linked to the time-rate-change of the magnetic moment *m*(*t*):

$$v_{suspension}\;\left(t\right)=\Sigma_{k=1}^{N}A_{k}\sin\left(2\pi kf_{0}t+\varphi_{k}\right)=-\frac{d\Phi \left(t\right)}{dt}=-\frac{1}{2\pi K_{pickup}}\frac{dm\left(t\right)}{dt},$$ (1)

with *K*_*pickup*_ a sensitivity coefficient in A·m^2^/V·s determined experimentally (described later). The instantaneous magnetization *M*(*t*) is thus determined by integration of the induced voltage $v_{suspension}\;\left(t\right)$:

$$M\left(t\right)=\frac{m\left(t\right)}{Vol}=\frac{K_{pickup}}{Volf_{0}}\Sigma_{k=1}^{N}\frac{A_{k}}{k}\text{cos}\left(2\pi kf_{0}t+\varphi_{k}\right),$$ (2)

where *Vol* is the volume of the suspension.

In the case of linear DMS, the magnetic moment can be defined by its complex susceptibility *χ*, the slope of the M-H curve, as $m\left(t\right)=Vol\chi H_{ext}\left(t\right)$. The susceptibility is projected into its real and imaginary components as $\chi =\chi^{\prime }-i\chi^{\prime \prime }=\left|\chi \right|e^{-i\Psi }$, with $\left|\chi \right|=A_{1}\frac{K_{pickup}}{Vol\left|H_{ext}\right|f_{0}}$ being the amplitude and $\Psi =\varphi_{1}+\frac{\pi }{2}$ being the phase.

The calibration coefficient *K*_*pickup*_, which captures the pick-up coil sensitivity, depends on the coil and the sample container geometries and is independent of the magnetic sample. This coefficient is determined by measuring the susceptibility spectra of magnetic particle suspensions using both a commercial calibrated AC susceptometer and the linear DMS.

## III. EXPERIMENTAL RESULTS AND DISCUSSION

Linear and nonlinear measurements are performed on two in-house magnetic nanoparticle suspensions obtained by thermal decomposition.^11^ The first suspension is made of PEG-coated iron oxide nanoparticles with ∼55 nm hydrodynamic diameter, ∼14 nm magnetic diameter suspended in water,^12^ while the second suspension is made of oleic-acid coated cobalt ferrite nanoparticles with ∼22 nm hydrodynamic diameter, ∼5 nm magnetic diameter suspended in 1-octadecene.^13^

### A. Linear DMS characterization

**FIG. 2.**
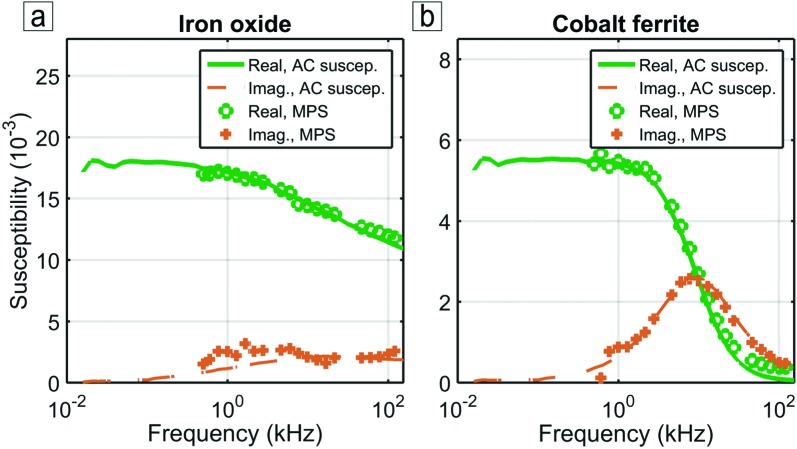
Iron oxide (a) and cobalt ferrite (b) particle characterization with linear DMS. Curves show very good agreement with measurements made by AC susceptometry.

Figure 2 shows linear DMS measurements for iron oxide and cobalt ferrite particles tested at 1 mT from 500 Hz to 120 kHz. The graphs show the in-phase real (green) and out-of-phase imaginary (brown) susceptibility component spectra as a function frequency. The markers are MPS measurements, while the solid and dashed lines are measurements from a commercial AC susceptometer (Dynomag, Acreo). We observe very good agreement between the two instruments for both particles, but because the sensitivity of the inductive sensing method decreases linearly with the frequency, we observe some discrepancy at lower frequency.

The iron oxide susceptibility spectrum (Figure 2 a) is characteristic of particles relaxing predominantly by the Néel mechanism. The in-phase component remains significantly higher than the out-of-phase component. The cobalt ferrite susceptibility spectrum (Figure 2 b) is characteristic of particles relaxing predominantly by the Brownian mechanism. At low frequency, the in-phase component plateaus with a zero out-of-phase component. Around 9 kHz the in-phase susceptibility drops dramatically while the out-of-phase peaks to its maximum. Finally, both components asymptote to zero as the particle rotations do not respond to overly high frequency excitation field.

### B. Nonlinear MPS characterization

**FIG. 3.**
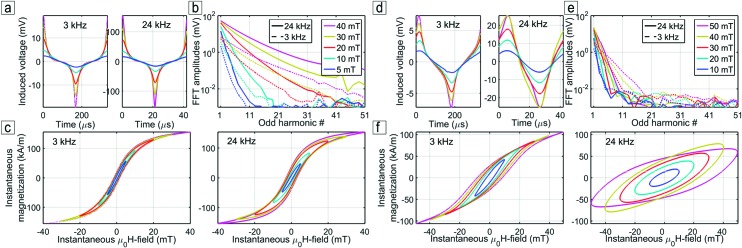
Iron oxide (a,b,c) and cobalt ferrite (d,e,f) non-linear MPS characterization at 3 kHz and 24 kHz, from 5 to 50 mT: time-varying induced voltage (a,d), voltage FFT spectra (b,e) and hysteresis curves (c,f).

Iron oxide and cobalt ferrite nonlinear MPS measurements at 3 kHz and 24 kHz from 5 to 50 mT are presented in Figure 3, displaying the measured time-varying induced voltage (a,d), the voltage FFT spectra (b,e) and the corresponding dynamic magnetic hysteresis curves (c,f). In both cases, the induced voltage increases linearly with magnetic field amplitude. At low excitation fields we see that the voltage response is almost sinusoidal, with few odd harmonics and an almost linear instantaneous magnetization response. However, at high excitation field strength, the voltages change abruptly, resulting in slow decaying FFT spectra and magnetization saturation.

For the iron oxide sample, the responses at 3 kHz and 24 kHz are very similar, consistent with Néel relaxing particles with characteristic peak frequency that is much higher than the frequency window of the measurement. The voltage induced at 24 kHz is 8 times higher than at 3 kHz, with the ratio corresponding to the frequency ratio as explained by the magnetic induction phenomena. Moreover, the voltage FFT spectra between 3 kHz and 24 kHz decay at similar rates, providing evidence of induced voltages with the same time variations.

For the cobalt ferrite sample, the responses at 3 kHz and 24 kHz are completely different, consistent with Brownian relaxing particles with a 9 kHz peak frequency that lies between the two studied frequencies. On one hand, the behavior at 3 kHz is similar to the response of the iron oxide suspension, albeit presenting broad voltage peaks and faster voltage FFT decays. On the other hand, the behavior at 24 kHz changes dramatically compared to 3 kHz, which is characteristic of a frequency that exceeds the inverse of the particle’s Brownian relaxation time: the voltage switches are even less sharp, the voltage FFT decays more rapidly than at 3 kHz and the instantaneous magnetization reaches a lower magnetization. The two sets of measurements show a shift between two regimes as supported by the linear DMS measurements on Figure 2 since the 9 kHz out-of-phase peak is located between the two frequencies.

**FIG. 4.**
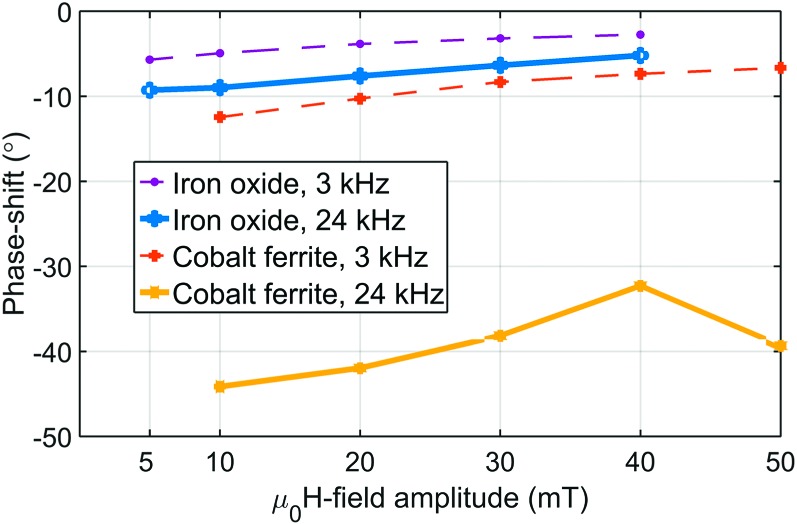
Nonlinear MPS phase shifts obtained for iron oxide and cobalt ferrite.

Figure 4 compares the phase shift between the instantaneous magnetization and the magnetic field for all field amplitudes and frequencies. While increasing the magnetic field amplitude, the magnetic torque increases, shortening the relaxation time and diminishing the phase shift. Increasing the frequency increases the hydrodynamic torque and thus the phase shift. The phase shift change from 3 kHz to 24 kHz is relatively small for iron oxide, but dramatically larger for cobalt ferrite, supporting the dramatic behavior change with the frequency. The direct effect of the phase shift is the appearance of a magnetization curve opening, i.e. a non-zero dynamic remanence and coercivity in the dynamic magnetization curve. As a consequence, curve openings in Figures 3c,f are larger at 24 kHz than at 3 kHz and larger for cobalt ferrite than for iron oxide.

## IV. CONCLUSION

This paper presents the design and validation of a magnetic particle spectrometer to study the linear and nonlinear behavior of magnetic nanoparticle suspensions. The setup design is detailed along with the post-processing and calibration procedures. Linear DMS measurements at 1 mT were realized in a wide frequency range (0.5–120 kHz) showing good agreement with a commercial AC susceptometer. Nonlinear MPS measurements require resonant and matching circuits to apply 50 mT at discrete frequencies from 3 kHz to 24 kHz. The pick-up coil system design and the feed-through cancellation procedure allows for fine measurements up to 50 harmonics. The two MPS modes are tested for iron oxide and cobalt ferrite suspensions, which exhibit very different magnetic relaxation behaviors. The measured time varying induced voltage, the voltage FFT, and the reconstructed instantaneous magnetization analysis were used to assess the magnetic suspension rotational dynamics and to investigate their relaxation and saturation effects.
